# “If your mother does not teach you, the world will…”: a qualitative study of parent-adolescent communication on sexual and reproductive health issues in Border districts of eastern Uganda

**DOI:** 10.1186/s12889-023-15562-6

**Published:** 2023-04-11

**Authors:** Patricia Ndugga, Betty Kwagala, Stephen Ojiambo Wandera, Peter Kisaakye, Martin K. Mbonye, Fred Ngabirano

**Affiliations:** 1 College of Business and Management Sciences (CoBAMS), School of Statistics and Planning, Department of Population Studies, Makerere University, Kampala, Uganda; 2 Labor and Social Development (MGLSD), Ministry of Gender, Kampala, Uganda

**Keywords:** Adolescent, Parent, Communication, Sexual and reproductive health, Barriers, Facilitators, Border, Uganda

## Abstract

**Background:**

Adolescents experience a host of sexual and reproductive health (SRH) challenges, with detrimental SRH and socio-economic consequences. These include early sexual debut, sexually transmitted infections including HIV/AIDS, teenage pregnancy, and early childbearing. Parent-adolescent communication about SRH has significant potential to reduce adolescents’ risky sexual behaviors. However, communication between parents and adolescents is limited. This study explored the facilitators and barriers to parent-adolescent communication about sexual and reproductive health.

**Methods:**

We conducted a qualitative study in the border districts of Busia and Tororo in Eastern Uganda. Data collection entailed 8 Focus Group Discussions comprising of parents, adolescents (10–17 years), and 25 key informants. Interviews were audio-recorded, transcribed, and translated into English. Thematic analysis was conducted with the aid of NVIVO 12 software.

**Results:**

Participants acknowledged the key role parents play in communicating SRH matters; however, only a few parents engage in such discussions. Facilitators of parent-adolescent communication were: having a good parent-child relationship which makes parents approachable and motivates children to discuss issues openly, a closer bond between mothers and children which is partly attributed to gender roles and expectations eases communication, and having parents with high education making them more knowledgeable and confident when discussing SRH issues with children. However, the discussions are limited by cultural norms that treat parent-child conversations on SRH as a taboo, parents’ lack of knowledge, and parents busy work schedules made them unavailable to address pertinent SRH issues.

**Conclusion:**

Parents’ ability to communicate with their children is hindered by cultural barriers, busy work schedules, and a lack of knowledge. Engaging all stakeholders including parents to deconstruct sociocultural norms around adolescent SRH, developing the capacity of parents to confidently initiate and convey accurate SRH information, initiation of SRH discussions at early ages, and integrating parent-adolescent communication into parenting interventions, are potential strategies to improve SRH communication between parents and adolescents in high-risk settings such as borders.

## Introduction

Globally, adolescents aged 10–19 years in sub-Saharan Africa (SSA) bear a disproportionate burden of sexual and reproductive health (SRH) challenges [1–3]. The region accounts for the highest rates of early marriage, adolescent pregnancy, unsafe abortions, complications during pregnancy and childbirth [4], and HIV transmission [5], contributing to high morbidity and mortality rates. The World Health Organization (WHO) estimates that one in 20 adolescents contract a sexually transmitted infection (STI) each year [6].

According to the 2016 Uganda Demographic and Health Survey (UDHS), teenage pregnancy stands at 25%, with rural areas (27%) having higher rates than urban areas (19%)[7]. Teenage pregnancy statistics worsened during the 2020–2021 COVID-19 era compared to the pre-COVID-19 period. In some districts such as Namisindwa and Amudat, teenage pregnancy increased by over 50% during the COVID-19 pandemic that disrupted provision of SRH services [8].

Access to timely sexual and reproductive health information and services is fundamental in improving SRH outcomes for adolescents. Studies elsewhere have shown that media, peers, teachers, and health workers are the main source of SRH information among adolescents [9–11]. However, information from peers and media may be incorrect leading to misrepresentation making adolescents vulnerable to poor sexual and reproductive health outcomes.

Some studies have shown that adolescents also prefer obtaining SRH information from parents [12–15]. Thus, parent-adolescent communication on SRH issues has the potential to prevent children’s involvement in risky sexual behaviors and empower them with decision-making skills [16–18]. Okigbo, Kabiru [19] in their study among adolescents living in Kenyan slums noted that male adolescents who reported communication with their mothers were less likely to transition to first sexual intercourse compared to those who did not. Despite these benefits, such important conversations seldom occur in many settings in SSA.

In Uganda, just like most SSA countries, several factors prevent discussions between parents and children. Parents are generally uncomfortable discussing sex-related issues with their children and have limited knowledge and skills to communicate effectively on SRH issues [20–22]. A qualitative review on barriers to parent-child communication on SRH issues in East Africa found gender differences, level of education, parents’ occupations, religion, and social-cultural norms as key barriers to communication about SRH [23].

Previous studies on parent-child communication about SRH in Uganda have targeted adolescents in a school setting [20], only considered parents perspectives [24] and among very young adolescents (10–14 years) [25]. Furthermore, these studies have only been conducted in urban and peri-urban settings in non-border settings [20, 25]. To the best of our knowledge, there are no published studies on parent-adolescent communication on sexual and reproductive health in border districts of Uganda where the population is very mobile and highly engaged in busy commercial activities [26, 27] which provide increased opportunities for engaging in risky sexual behaviors. Border areas have a higher HIV prevalence compared to non-border areas [28–30]. Mobile populations at the borders may lack access to SRH services and those working away from home usually engage in casual sexual relationships while traveling.

This study aimed to fill this gap by assessing the practices, barriers, and facilitators of parent-adolescent communication about SRH in two Eastern Uganda border districts. In this study, sexual and reproductive health refers to a wide range of topics, including abstinence, methods of contraception, HIV/AIDS and other STIs, unwanted pregnancy, condoms, sexual intercourse, and menstruation.

## Methods

### Study design and setting

Data collection was conducted between 2nd and 18th May, 2021. We used a qualitative research design to gain a deeper understanding of the facilitators and barriers of parent-adolescent communication on SRH issues in two border districts of Busia and Tororo, located in Eastern Uganda.

According to the 2014 National Population and Housing Census [31], Busia and Tororo have a population of 323,662 and 517,082 respectively. Busia and Tororo share borders with Kenya and host the busiest ports of entry in Uganda. The predominant ethnic groups in the study districts are the Samia and Itesot in Busia, and the Japhadola and Itesot in Tororo. They have a diverse population comprising truck drivers and other transporters, cross-border traders, sex workers, border officials, border town residents, and tourists/visitors. As such, the situation in the border districts of Uganda is dire owing to the cross-border trade and transient populations, which elevate the risk of poor SRH outcomes. Residing in border areas present unique economic and social challenges such as extreme poverty, family separation (parents working in neighboring countries) which may result into adolescents seeking high risk jobs such as vending and cross-border trading. This exposes them to multiple vulnerabilities, such as transactional sex, having multiple sexual partners and high HIV vulnerability [32–35]. Owing to the cross border work opportunities, many mothers cross the border for domestic employment for extended periods of time, thus, there is an increasing number of single parent households in Busia and Tororo.

The main religion in the area is Christianity, while the main economic activities are cross-border trade, small-scale business, subsistence farming, sand mining, stone quarrying, fishing and gold mining. Communities in Busia and Tororo practice a popular funeral fundraising gathering called “Disco Matanga” loosely translated as ‘disco at a funeral’ whereby during a funeral, adults fundraise for the burial expenses for the departed. This fundraising is done in the night with loud music playing all night which attracts both children and adults. Reports indicate that many children (as young as four years) attend this gathering unaccompanied by parents.

### Study population

The study population were parents of adolescents aged 10–17 years, adolescent boys and girls aged 10–17 years, and key informants. While the World Health Organization classifies adolescents as those aged 10–19-year-old, our study focused on 10-17-year-olds due to the unique legal and policy implications faced by this age group as compared to older 18–19 year-olds who are of legal age of consent according to the Uganda legal consent age of 18. Parents in this study referred to a biological mother/father or female/male caregiver of the adolescent (aged 10–17) who must have lived continuously with the adolescent for at least one year prior to data collection.

### Sampling

A multi-stage stratified sampling design was used. From each district, two subcounties were randomly selected. A total of four sub-counties were selected – two from each district. From Tororo, Malaba TC (urban) and Mella subcounty (rural) were selected. In Busia district, Dabani (peri-urban) and Buhehe (rural) were selected using computer random numbers using Microsoft Office Excel programme. From each sub-county, two parishes were randomly selected. From Malaba TC, Obore and Amagoro parishes were selected. From Mella, Apokor and Mella parishes were selected. From Dabani, Buyengo and Dabani parishes were selected. From Buhehe, Bulwenge and Buhasaba parishes were selected. Finally, a total of 10 villages were selected using simple random sampling from these parishes. From each village, purposive sampling was used to identify households with parents who have children aged 10–17 years. Only one child and one parent was randomly selected from each household for interview. Parents with adolescent children were identified with the help of local leaders in the community.

### Data collection methods

Focus group discussions (FGDs) and key informant interviews (KIIs) were the methods used for qualitative data collection. FGDs were used to capture a wide range of views and enable interaction between participants with differing experiences regarding parent adolescent communication which provided greater insight into attitudes, perceptions, beliefs and practices. The FGD guides were translated into Lusamia, Japhadola and Ateso, the predominant local languages.

We conducted 8 FGDs with fathers (n = 2), mothers (n = 2), boys (n = 2) and girls (n = 2) adolescents in separate specific gender- groups. The FGDs were disaggregated by sex to allow for free expression of views during the discussion of potentially sensitive issues. We conducted four FGDs in each district (Table 1).

**Table 1:** Summary of FGDs conducted in Busia and Tororo

	Busia		Tororo	
	FGD		FGD	
**Parents**				
Fathers only	1		1	
Mothers only	1		1	
**Adolescents**				
Boys only	1		1	
Girls only	1		1	
Total	**4**		**4**	

Owing to the observation of the COVID 19 standard operating procedures (SOPs), each FGD had six participants and lasted approximately 1 h and 30 min. All FGDs were conducted by a moderator and a note-taker. Consent from participants was sought to audio record the discussions.

The interviews were conducted by the research team members (PN, BK, SOW & PK) along with a team of 6 youthful male and female research assistants (RAs). The research assistants used were below 25 years of age. This was to avoid much age disparity between the research assistants and the participants in order for the respondents to communicate freely. Given the sensitivity of this topic and cultural traditions in the study contexts, participants were interviewed by an interviewer of the same sex. Male research assistants moderated FGDs which comprised of male participants and females did the same for FGDs of females. The RAs had bachelor’s degree qualification in social sciences with significant experience in working with children, collecting data on sensitive topics, including sexual behavior, and were proficient in the local languages (Lusamia, Japhadola and Ateso). All research assistants were trained on research ethics, principles of qualitative data collection, and the study procedures and instruments.

To ensure privacy, the FGDs were held in an open space (within the household compound) which offered privacy during the interviews with children so that their responses are not heard by their parents. FGD participants were provided with refreshments as compensation for their time.

Additionally, 25 KIIs (13 in Tororo and 12 in Busia) were conducted with four categories of key informants that work with children: Non-Governmental Organizations (NGOs), Community Based Organizations (CBOs), officers from the District Local Government (DLG), and community leaders consisting of religious, cultural and local leaders. Key informant interviews elicited information on parents’ knowledge, attitudes, and practices about parent-child communication (PCC) on SRH; determinants of PCC; examine the facilitators and barriers of PCC; and identify parents’ and children’s preferred approaches to PCC on SRH in eastern Uganda. The KIIs were conducted in English using a semi-structured interview guide. The duration of the interviews ranged between 30 and 45 min.

### Data collection tools

Three semi-structured interview guides (Key informant guide, FGD guide for parents, FGD guide for adolescents) were developed. The questions were based on a review of the literature, field experience, and research objectives. Areas explored included: adolescents’ and parents’ knowledge, attitudes, practices and preferred approaches to parent child communication, barriers and facilitators of parent adolescent communication, frequency and timing of SRH communication, experiences and perceptions in SRH communication, preferred sources of SRH information.

### Quality control and assurance

We recruited competent interviewers who had experience in working with children and could speak the local languages. Training of interviewers was conducted to ensure detailed understanding of objectives, process, and output requirements. Close supervision of research assistants by the research team ensured that data is collected in a manner that maintained data integrity. The tools were translated from English to the local languages by a language professional and back translated to English to ensure conceptual equivalence and cultural sensitivity. The tools were pretested to check for accuracy in a neighboring district (Namayingo) among population groups similar to the study population before collection of data. Furthermore, the use of a tape recorder, careful probing, and interviewing up to data saturation were activities done to ensure dependability.

### Data management and analysis

Following fieldwork, audio files from interviews were transcribed then translated into English. Handwritten notes were used to supplement information gaps from the audio-recorded transcripts. Transcripts were thematically analyzed using an inductive approach. Two members of the study team (PN & BK) read and re-read transcripts to become familiar with the data. Transcripts were annotated with initial codes relevant to the study objectives which formed the initial coding frame. The codebook was discussed and agreed upon by all members of the research team. The developed themes and sub-themes were then entered as codes into NVIVO 12 software. The two researchers (PN & BK) independently coded transcripts and met regularly to review for consistency. Discrepancies were resolved through discussion and input from the other researchers. New codes were added as they emerged and analysis continued until no new codes were identified.

## Results

### Study participants’ background characteristics

Tables 2 and 3 show the background characteristics of the study participants. There were equal number of males and females engaged in all FGDs. All parents were aged between 28 and 55, 23 were married while 1 was divorced. The adolescents were aged between 10 and 17 years and all were not married. We selected 25 Key informants, 11 females and 14 males. The participants comprised of 8 community leaders (6 religious leaders, 2 cultural leaders), 11 from the District Local Government, 2 from community-based organizations, and 4 from Non-Government organizations. Their ages ranged between 25 and 75 years. Most of them were married and belonged to the Christian faith.

**Table 2:** Background characteristics of the FGD participants

	Adolescents (N)	Parents (N)
**Sex**		
Male	12	12
Female	12	12
**Age**		
10–14	8	
15–17	16	
28–45		18
46–55		6
**Education level**		
None		4
Primary	10	8
Secondary	14	11
Tertiary		1
None		
**Marital Status**		
Single	24	
Married		23
Divorced		1

**Table 3:** Background characteristics of the Key Informants

Characteristic	Frequency
KI categories	
NGO	4
District Local Government	11
Community Leaders*	8
**Sex**	
Male	14
Female	11
**Age**	
36–45	5
46–55	10
56–75	7
**Education level**	
Secondary	3
Diploma	6
Tertiary	16
**Marital Status**	
Single	4
Married	21
**Religion**	
Born again	7
Anglican	8
Catholic	4
Muslim	6

*These included religious leaders, cultural leaders, and local leaders

### Parent-adolescent communication practices in Busia and Tororo border districts

Participants were asked whether discussions on SRH between parents and children occur within the community. They mentioned that in the past, parents were not expected to discuss SRH with their biological children. This role was delegated to grandparents, paternal aunties and uncles. However, many participants recognized that the times have changed due to consequences of HIV/AIDs, high teenage pregnancy, influence of media and busy work schedules. Majority of the study participants acknowledged the key role parents play in communicating SRH matters with adolescents, however, they reported that only a few parents discuss SRH with their children.

*Parents would be the best people to communicate to their children, but few parents do that. So they play a very small role (Mother, FGD, Busia).*

*I would say the parents have not done their part. …if the parents would step in and take lead, we wouldn’t be having this outburst of teenage pregnancy… (KI, NGO, Tororo).*

Adolescents reported that most SRH discussions were spontaneous and often triggered by: parents perceiving the child’s behavior as risky, signs and symptom of disease among adolescents or an unpleasant occurrence in the community (such as a neighbor’s teenage daughter falling pregnant).

*When you find your daughter at the truck parking yard [parking yard for cargo trucks at the border] then you should talk right there. That means she is spending time with truck drivers who will make her drop out of school (KI, DLG, Busia)*

*…if I have a swelling on my penis, I tell my mother what I am feeling (Boy, FGD, Tororo)*

Examples of behaviors that parents perceived to be risky were: indecent dressing, being part of “bad” peer groups, movements late in the night, and associating with the opposite sex. Parents and adolescents were asked to describe the topics that are discussed when they have conversations on SRH matters. The responses from parents and adolescents were similar. The discussions mainly focused on abstinence from premarital sex, pubertal changes, and relationships with the opposite sex, STIs including HIV/AIDs, teenage pregnancy and risks associated with late night movements.

*Some talk about HIV/AIDS especially on ways how they can prevent contracting it. For the older children, parents tell them about issues of sleeping with boys, about STIs or getting pregnant (KI, Community leader, Tororo)*. Parents were asked if there are sex-related topics that should never be discussed with adolescents. A few parents were of the opinion that there were no forbidden topics. Topics on “sexual intercourse” and contraceptive use especially condom use were rarely discussed.

...*sex is difficult to talk about…telling them that ‘if you are to have sex, do this and that’ most parents cannot say that to the children. (Mother, FGD, Busia)*

They felt that adolescents need to understand sexual and reproductive health issues and implications of making wrong decisions. This information, they believe, should be provided to adolescents at home so that they are not misinformed by strangers.

When asked about the age they thought was right to talk to their children about sex, most said the discussion should start once the child reaches adolescence. Others mentioned that it was when girls start menstruation (at about 10 years), whereas among the boys, it was at a later age (< 13 years). However, a few parents felt that these discussions should start as early as 5 years among both boys and girls. Gender differences existed in parent adolescent SRH communication with more caution targeting girls as compared to boys.…Mothers are expected to speak to girls and fathers to the boys. *“It’s very hard for the daughter to tell the father her secrets (Father, Busia, FGD).* Some few participants mentioned that SRH education is almost nonexistent among boys and their parents. They believe boys can thrive without guidance or direction.

*…boys grow like weeds. They don’t get pregnant; if he is unlucky he is imprisoned for impregnating a girl (Father, FGD, Tororo)*

### Facilitators of parent-adolescent communication on SRH issues

The following themes emerged as facilitators: good parent-child relationship, the role of the mother, and education level of the parent.

#### I. Good parent-child relationship

Participants mentioned that when there is a good parent-child relationship, defined as the ability of parents and children to approach each other and discuss any SRH issues openly. Adolescents who are close to their parents are motivated and able to initiate discussions on SRH matters. On the other hand, when the relationship is poor there is no communication.

*If the child and parent are free with each other, it is easy for them to talk. You may be willing to talk to her, but she isn’t free with you, she will end up seeking advice from the neighbors or friends just because she is not free with you. (Mother, FGD, Busia)*

*Children will always open up to a person who is friendly, welcoming and does not discriminate. Someone who has time for them they will always open up to that person. (KI, NGO, Tororo)*

*Parents should be able to have this free conversation between themselves and the children to make the children gain their trust so that they can be able to tell them in case anything happens. (KI, DLG, Tororo)*

Other participants reported that having a good parent child relationship resulted from being a suitable model for the adolescents which was an enabler for parent-child communication on SRH matters. Children felt that they should receive guidance from parents with a good conduct.

*You [parent] are telling them to abstain, yet you have very many children with different fathers. So sometimes they feel they should listen to someone who has a good record (KI, DLG, Busia)*

#### II. Role of the mother

Participants’ narratives suggest that mothers were key in influencing parent-child communication. Given their gender role as care givers and home educators, mothers dominated parent-child communication on SRH matters. Mothers were considered close to their children and spent longer periods of time with them than fathers. Mothers perceived themselves to be better prepared and more approachable by their children.

*Mothers are always available to talk. They are gentler when dealing with us (Girl, FGD, Busia)*

*Most of the children associate with their mums, so, it’s easy for them to tell their mum what is happening to their bodies. (KI, CBO, Tororo)*

Others referred to a mother as one who can be trusted with secrets and usually finds ways of helping children to address SRH issues. Mothers were also considered sympathetic and less harsh which made them more approachable and had more experience in discussing SRH issues with their children.

*When you tell her [mother] about something, she will not tell the neighbors about your issue, she will keep it a secret. (Girl, FGD, Tororo)*

Study participants noted that many fathers abdicate their roles of communicating about SRH to the mothers. Fathers were too strict, unapproachable, unavailable and too be busy to listen to children. They send the children, including boys to the mothers for counsel which is also a driving factor as to why mothers are most often spoken to.

*Most children are afraid of their father, even a boy who wants a new book, he will still go to the mother... They fear fathers because they are hostile to them…(Boy, FGD, Busia)*

#### III. Education level and exposure of the parent

Parents with higher levels of education were better positioned to communicate with their children about SRH compared to those with less-education. Such parents had better communication skills and were more knowledgeable and able to respond to technical SRH questions raised by children. Less-educated parents may feel uncomfortable talking to their children about SRH.

*Educated parents have information so they can explain some of these things to their children. When I look at a parent who is a school dropout; what information will he or she give to a child? (KI, community leader, Tororo)*

### Barriers to parent-child communication on SRH

#### I. Cultural norms

Cultural norms were the most commonly reported barrier to parent-child SRH discussions. These made it unacceptable for parents and children to openly discuss SRH matters. Many of these parents reported that parents in their setting did not discuss sex matters with their children because it is a taboo thus, it is an abomination to speak about sex with your child. They perceive SRH matters to be private and that children would come to know about these issues automatically without any discussion with parents.

*…They feel it is not right to speak to your child about sex. They still think sex is a bad thing, it is private. It should be discovered by you who is having it and it’s not a matter of discussion. Yeah. Yeah. It is a taboo. (KI, CBO, Tororo)*

Closely associated to cultural norms, was embarrassment as an inhibitor of communication both on the part of children and on the side of parents. Majority of the parents revealed that embarrassment, shame and awkwardness kept them from initiating the discussion with their children. This was corroborated by the adolescents. Parents felt “embarrassed” talking about SRH with their children because culture labels SRH topics as a taboo.

*They also fear to approach us. Unless you find one who is very brave to confront you, most of them are shy just like us the parents. She will not come and tell you about the man who is trying to convince her into a relationship. (Mother, FGD, Busia).*

. *..Some time back, my mother wanted to give me condoms, but she was fearing to tell me. …she told me that I want to give you something, She placed it at a table and just directed me …there is something there …you go and pick it, eeeeh (Boy, FGD, Busia)*

*…things like boys seducing you is your secret because you feel shy sharing such information with your parent and so you keep it as your secret (Girl, FGD, Busia)*

Given the culture limitation, parents and children face difficulty in discussing SRH issues resulting into parents speaking to the children in parables, which limits comprehension. For example, the expression *“If your mother does not teach you, the world will…”* that was made by a girl from Tororo meaning that if morals, values, and good character are not imparted at home, then you will learn from hard knocks or problems that result from a lack of or neglect of instruction. Another young girl reported that her mother keeps saying: *“…this world is very bitter and very dangerous you have to be very careful with your life” (Girl, FGD, Busia)*. Another parent reported *“…the world has gone wild, sicknesses are coming like water “(Father, FGD, Tororo)*.

Most of the parents are reserved and tend not to discuss in details they just caution the children, for example, “*I don’t want to see that you move with the boys. I don’t want to see you getting pregnant, I don’t want to see you in the company that may mislead you, so that is the much they can open up with their children” (KI, CBO, Busia)*.

They believe that words associated with SRH are obscene and would expose children to inappropriate information that could result in experimenting with sex or encourage early sexual activity. “*It’s like you can’t talk about sexual relationship it is like umm you are encouraging them to try out … Umm the word sex is culturally forbidden” ( KI, DLG, Tororo)”*

*…Do you want to make me old [grandmother] yet I am still young? (Mother, FGD, Tororo)*

*…they fear* telling their children to use condoms because they feel the child will engage in sex knowing that it was the *mother who advised her. (Mother, FGD, Busia)*.

Due to the sensitivity of SRH issues, some parents revealed that they would involve a “third party” usually a paternal aunt, uncle or teacher to discuss SRH matters with the adolescents. Some parents expect teachers to inform or teach children about SRH, yet teachers rarely do so. A key informant from Busia reported that the moment he learns that SRH has been integrated into the curriculum, then many parents including himself, will not discuss SRH with his children. They explained that children are free with the teachers.

... *You can feel ashamed discussing these things with your parents but if the senior woman teacher talks, you can’t feel ashamed (Girl, FGD, Tororo)*

#### II. Busy parents

Parents’ occupations determined the available time they spend with their children. Parents increasingly prioritized the demands of employment over child care. Many participants reported that parents were too busy to dedicate time to talk to their children about SRH. Owing to work demands, some parents do not live with their children. Concerns were expressed about absent mothers who travel for domestic employment elsewhere (mainly to neighboring Kenya) for extended periods of over a year, leaving fathers who give little time to children. Other parents leave very early in the morning and return late at night when they are tired, and the children are asleep.

*Because we the parents are busy, we leave very early in the morning and come back at night. And even when we come back we say we are tired, we don’t have time to talk to children mmmh (Mother, FGD, Tororo)*

*…if the parent comes home late, there is minimal social interaction with the children (KI, Community leader, Tororo)*

#### III. Parents’ lack of knowledge

Parents felt they lacked the knowledge, appropriate skills, and approaches to talk to their children about SRH, making it challenging to initiate a conversation. Some parents expressed the need for well packaged and age appropriate SRH information to enable them to address these topics. Parents reported that this lack of knowledge created a lack of confidence, making it difficult to find the courage to start a discussion with their child. A few parents had the knowledge but lacked the confidence to discuss SRH matters with children. Children also mentioned that their parents were less knowledgeable about SRH. This perception kept them from initiating a conversation with their parents.

*…children nowadays know a lot more than what we the parents know. You might tell her something thinking it is new yet she knows much more than you do (Mothers, FGD, Busia)*

*Some parents do not have information. We are told to talk to our children about these things, but, how should we begin, we don’t know what to say (FGD, Fathers, Busia)*

…*I think some parents do not have proper information and others don’t know how to talk to children (Girl, FGD, Tororo)*

Some participants raised concerns of excessive alcohol consumption and drug abuse among both parents and children which is a serious challenge that hinders SRH talk. Parents, especially fathers, get drunk and are unable to guide adolescents to make wise SRH decisions

*Fathers report to a Malwa [local brew] joint at six am … They go back home after 9 or 10 p.m. just to sleep. They do not have time for children (KI, NGO, Tororo)*

*Some don’t talk to their children b*ecaus*e they are drunk all the time (Boy, FGD, Tororo)*

*…you find 10 year olds with sachets of Waragi [local brew] and such a child once he or she is drunk you cannot bring up a conversation on SRH (KI, DLG, Tororo)*.

When most parents talk to their children, they use authoritative, reprimanding language, especially when they observe cases of teenage pregnancy in the community. Respondents reported that fathers are tough, harsh, and instill fear in the children. Some threaten and use corporal punishment to discipline or warn adolescents concerning inappropriate behavior, especially sexual activity. Owing to the harsh approach used by fathers, most girls and some boys prefer sharing their concerns with mothers. One girl from Tororo reported that when she asked her father for a mathematical set, he angrily told her, “ *…but you are a girl, can’t you think of a way of making that money to buy the set?”* One boy said:

*There are some fathers you tell your concerns, but they just start quarreling, accusing you of being spoilt. This creates fear and makes children keep* quiet. (Boy, FGD, Busia)

As a result, many children do not communicate with their parents. They tend to be shy, and fear punishment. They detest the harsh language used by some parents and only communicate in case of a crisis. The children report that some parents curse the children and tell them not to revert to them in case of problems. This approach can be counter-productive and contributes to rebellion and early marriages. In addition to attributing early marriages to the inability to cater for children’s needs, a mother confirmed the children’s observations:

*Some girls get married at an early age because of us parents. Sometimes, we are very hostile and yet if you don’t take good care of the children…The child will run away and get her own home (Mother, FGD, Busia)*

*Unfortunately with our parents the communication I should say is poor because it is about shouting, it’s more of discipline, …it’s about seeing a girl with a boy then they begin beating the child, shouting that if you get pregnant I will chase you away, I will cut your head off yeah so it’s more of like disciplinary action (Girl, FGD, Tororo).*

Many parents indicated that harsh language is used in an endeavor to make the children understand the severity of the issues at stake. Such language is also used when the children are disobedient, for instance, when they break household curfew regulations. This approach is expected to ensure that children do not start or continue with inappropriate behavior.

*I think you need to be harsh and threaten them with police involvement because that is the only way they understand. Whatever the child does comes back to you the mother….So in a way we need to be harsh to them so that they take whatever we tell them seriously.* (Mother, Busia, FGD)

*You need to be tough with them because if you bring up such issues in a joking way, then she will take it lightly as a joke. That said, there are some sensitive issues that you ought to bring out in a polite way to earn their respect and confidence like things to do with their menstrual periods for the very first time. (*Mother, Busia, FGD)

## Discussion

To the best of our knowledge, this is the first qualitative study to assess the facilitators and barriers of parent-adolescent communication on sexual and reproductive health in a Ugandan border setting. We captured the views of 10-17-year-old adolescents, parents, and key Informants in two border districts located in Eastern Uganda. The findings highlight several important points that are useful for designing interventions to improve parent-adolescent communication among parents and children.

This study found that only a few parents had SRH discussions with their children. This finding is in line with those of previous studies from sub-Saharan Africa [11, 13, 30–32]. In their study among adolescents aged 13–17 years in Nigeria, Mbachu, Agu [31] found that majority of adolescents had never discussed sex-related matters with their parents. Among those who engaged in SRH discussions, these mainly focused on abstinence and HIV/AIDS. This finding is also consistent with the findings of Mbachu, Agu [31], Wamoyi, Fenwick [33], and Seif and Moshiro [34]. A possible explanation might be that border settings characterized by cross-border movements and trade tend to have disproportionate rates of prostitution, violence and HIV/ STIs prevalence compared to populations in non-border areas [33–35]. Hence, parents’ emphasis on perceived effective preventive measures. Another possible explanation for this is that it is common for parents in conservative cultures to focus on abstinence-only messages given that contraception and sex are seen as a taboo. However, some adolescents and key informant reported that some of the discussions they had with parents focused on “how to contribute to the household income”. In other words, children were encouraged to engage in income generating activities. This is not surprising in this context with high levels of poverty [34] which requires involvement of both children and parents for household sustenance. Lack of proper SRH education reflects problems facing adolescents such as unprotected sex, unplanned pregnancies with unsafe abortions, HIV/STIs. The implications of this finding is that the parents should not be ignored in programs that wish to reduce adolescents’ risky sexual behaviors.

The major barrier to parent adolescent communication in this study was parent’s busy schedules due to pressures of work which hindered interaction between parents and children. A previous study by Mmbaga, Leonard [13] also confirmed that busy schedules hindered SRH discussions between parents and secondary school adolescents’ age 16–19 years in Tanzania. Another study conducted in rural and urban Uganda by Muhwezi, Katahoire [19] found that communication on SRH issues between parents and their children was also hindered by parents busy work schedules. Plausible explanations for this could be: Busia and Tororo are predominantly commercial towns with cross border trading in the context of high mobility [34]. Most men and women leave very early in the morning for work and many return late in the night. It is mainly women who cross the border (to Kenya) for work leaving behind husbands who rarely engage in SRH communication with children. Absent parents are less likely to have a close and trusting relationship with their children, which affects the communication process as documented by other studies in SSA [21, 36]. These work related stressors in a border setting leave little room for SRH discussions between parents and children. Abdication of parental roles leads adolescents to rely on peer influence and social media as the most common sources of SRH information. Unfortunately, studies have shown that information obtained from these sources is either incorrect or false. This is a major cause of early sexual activity, and consequently high rate of unwanted pregnancies and unsafe abortions among adolescents [19, 37].

Adolescents expressed a preference for discussions with mothers compared to fathers. Several studies in different contexts have demonstrated the key role mothers play in impacting children’s sexual and reproductive health decision making [18, 19, 38]. Mothers were described as being approachable and the discussions described as warm and open. A study among Jordanian and Syrian parents (mothers and fathers) of youth aged 15–19 years old indicated that mothers perceived themselves as being more approachable by their children [39]. Mothers were reported to have a closer bond with the children which is attributed to social and culturally formed gender roles and expectations. Achen, Nyakato [22] in their qualitative study examining the impact of gender norms and expectations on parent–child SRH communication in rural south-western Uganda argued that activities ascribed to girls such as doing household chores including cooking, cleaning and care giving roles ultimately prepare them to bond with children. Both girls and boys described SRH communication with fathers as non-existent, rare, difficult, and uncomfortable [39]. Presence of the mother has been highlighted elsewhere as having a higher impact on adolescent’s sexual behavior.

Many parents lacked confidence in their ability to discuss SRH matters with their children, attributing this, to their lack of relevant knowledge and also to their low level of educational achievement. Additionally, some parents lacked the communication skills, and were uncomfortable discussing sexual and reproductive health issues. For example, parents could not explicitly discuss sex with their children. Others had incorrect knowledge while others did not know what to tell the child which limited what they could communicate. A knowledgeable parent easily comprehends the importance of SRH communication and forms a favorable attitude to interact. Our findings are consistent with those of other studies among sub-Saharan populations [14, 20, 31, 38]. Others said they had not received any SRH education while growing up, therefore, found it difficult to confidently talk about issues they did not know much about. This highlighted a generational gap where parents draw on their own experiences growing up where such issues were not discussed within families. This study also found that parents avoided topics on condoms and contraception. This finding was also reported in a Tanzanian and South African study [11]. Parents’ failure to discuss contraception could arise from parents’ fear that such communication would be interpreted as an encouragement for sexual activity. Premarital sexual activity especially among adolescents is strongly discouraged in many SSA settings [40, 41] and thus discussions on SRH emphasize abstinence rather than contraception. Selective SRH topic discussions by parents violates adolescents’ right to comprehensive and accurate health information.

Findings show parents adopted a harsh and authoritarian approach to SRH communication, which made it difficult for children to openly discuss their SRH concerns. An open, loving and supportive relationship between parents and their children was a foundation for good parent-child communication. It was clear that adolescents who enjoyed a good relationship with their parents, especially their mother, were able to discuss any issues openly. However, these discussions often started late (onset of pubertal changes) when adolescents had already engaged in sexual activity. Late communication, particularly after adolescents have begun sexual activity is unlikely to influence decisions to abstain from sex or practice safe sex. A study by Downing, Jones [42] argued that the timing of parent-child SRH communication would be more effective if it takes place before sexual debut to reinforce protective factors. These findings underscore the urgency of enabling parents to initiate SRH communication with adolescents at younger ages in such a high risk sexual behavior (engaging in transactional sex, and having multiple sexual partners) context to avoid unwanted pregnancies and associated negative SRH outcomes.

Culturally it’s a taboo for parents to speak to their children about sexual and reproductive health matters. This finding is similar to previous studies [21, 30, 38] which also established that cultural norms do not allow parents to directly talk to their children about issues of sexual and reproductive health. This caused the parents and adolescents embarrassment to engage in SRH discussions. Ugandan culture ascribes paternal aunts (sengas) / uncles (kojjas) as the main source of SRH knowledge to adolescents [19, 43, 44]. This arrangement was possible in the old extended family environment, but as family became less extended, increased exposure to other sources of information such as schools, peers and social media, the cash economy and highly mobile population in border settings, the role of the paternal aunts and uncles has diminished. These findings suggest that cultural norms and conservative attitudes do not offer a friendly environment where issues of SRH could be honestly and openly discussed. As a result, adolescents are missing vital and beneficial SRH information and guidance.

## Conclusion

This study set out to explore the facilitators and barriers of parent-adolescent communication on SRH in two Eastern Uganda border districts. This study has shown that a good parent-child relationship, role of the mother and parents level of education were the main facilitators of parent adolescent communication. Conversely, parent-adolescent communication about sexual issues is reduced when parents are engaged in busy work schedules, cultural norms and having limited knowledge and skill to initiate SRH discussions with children. The results of this study highlight the unique sexual and reproductive health challenges faced by adolescents in border settings, placing them at a greater risk of poor SRH outcomes. This vulnerability creates an opportunity to engage all stakeholders including parents to deconstruct sociocultural norms around adolescent sexual and reproductive health, sensitizing and developing the capacity of parents, encourage initiation of SRH discussions at early ages and integrating parent-adolescent communication into parenting interventions, as potential strategies to improve SRH communication between parents and adolescents in high-risk settings such as borders.

### Limitations

A limitation of this study is that it was conducted among adolescents and parents living in border settings of Uganda, and findings may not be applicable to adolescents living in other urban or rural settings.

## Data Availability

The datasets used during the current study are available from the corresponding author on reasonable request.

## List of abbreviations

CBO: Community Based Organization
DLG: District Local Government
FGD: Focus Group Discussion
KI: Key Informant
NGO: Non-Governmental Organization
SRH: Sexual and reproductive health
STI: Sexually transmitted infection
SSA: sub-Saharan Africa
